# Pinosylvin Inhibits Inflammatory and Osteoclastogenesis via NLRP3 Inflammasome

**DOI:** 10.1002/advs.202501532

**Published:** 2025-06-10

**Authors:** Wei Zhang, Xiangbing Wu, Wenming Li, Haifeng Zhang, Yijun Wang, Jing Xu, Wenhao Li, Yi Qin, Zebin Wu, Gaoran Ge, Shujun Lv, Lu Mao, Liangliang Wang, Dechun Geng

**Affiliations:** ^1^ Department of Orthopaedics The First Affiliated Hospital of Soochow University No. 188 Shizi Street Suzhou Jiangsu 215006 China; ^2^ Department of Implant Dentistry Suzhou Stomatological Hospital Suzhou Jiangsu 215005 China; ^3^ Department of Orthopaedics Shanghai General Hospital Shanghai Jiao Tong University School of Medicine No.85 Wujin Road, Hongkou District Shanghai 200080 China; ^4^ Department of Anesthesiology Affiliated Hospital of Jiangsu University 438 Jie Fang Road Zhenjiang Jiangsu 212001 China; ^5^ Department of Orthopedics Hai'an People's Hospital Hai'an Jiangsu 226000 China; ^6^ Department of Spine Surgery Zhongda Hospital Southeast University Nanjing Jiangsu 210009 China; ^7^ Department of Orthopedics the Second People's Hospital of Changzhou the Third Affiliated Hospital of Nanjing Medical University Changzhou Jiangsu 213000 China

**Keywords:** inflammatory related bone loss, NEK7, osteoclastogenesis, Pinosylvin, pyroptosis

## Abstract

Pro‐inflammatory cytokines such as TNF, IL‐1, and IL‐6 trigger aberrant osteoclastogenesis and result in massive bone loss. During RANKL‐induced osteoclastogenesis, pyroptosis of macrophages/preosteoclasts acts as a pivotal mechanism for IL‐1β release, thereby promoting osteoclast maturation and bone resorption. In the current study, it is observed that Pinosylvin (PIN), a compound extracted from European red pine, selectively inhibits LPS‐ and RANKL‐induced release of IL‐1β effectively reducing osteoclastogenesis. Notably, PIN inhibits the assembly of NLRP3 and the cleavage of GSDMD, pro‐IL‐1β, and pro‐caspase‐1, suggesting its therapeutic effects are NLRP3‐targeted. Mechanistically, PIN blockes the NEK7/NLRP3 interaction, but not the NLRP3/ASC interaction, through its 3,5‐dihydroxy groups by binding to NEK7, thereby inhibiting subsequent pyroptosis and osteoclastogenesis. Importantly, PIN alleviates inflammatory bone loss due to estrogen deficiency, reduces cranial bone destruction from local LPS injections, and improves survival in LPS‐induced septic mice. This study uncovers the specific mechanism behind PIN's potent anti‐inflammatory effects and identifies a new therapeutic target for NLRP3‐driven diseases.

## Graphical abstract

Pinosylvin blocks LPS and RANKL induced NEK7/NLRP3 interaction, inhibiting NLRP3 inflammasome oligomerization and assembly, reducing IL‐1β maturation and GSDMD cleavage, and improving the inflammatory microenvironment and abnormal osteoclastogenesis.

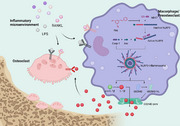

## 1 Introduction

Inflammation is a major, often overlooked, cause of both local and systemic bone loss, which can ultimately lead to patient disability or death.^[^ ^1^ ^]^ The negative balance of bone remodeling seen in conditions like osteoporosis, periprosthetic osteolysis, inflammatory bowel disease, periodontitis, rheumatoid arthritis, and other acute and chronic inflammatory diseases is primarily driven by inflammatory cytokines such as IL‐1β, TNF‐α, and IL‐6.^[^ ^1^, ^2^ ^]^ These cytokines promote abnormal osteoclastogenesis while inhibiting osteoblast function. Several treatments for inflammatory bone loss target the imbalance in bone remodeling, including bisphosphonates, denosumab, teriparatide, and romosozumab, with varying degrees of success. Importantly, alleviating primary or secondary systemic inflammation is crucial for treating inflammatory bone loss. Clinically, glucocorticoids are the primary drugs used to reduce systemic inflammation; however, they are also a significant cause of femoral head necrosis and osteoporosis.^[^ ^3^ ^]^ Therefore, exploring the mechanisms behind the inflammatory cytokine storm in these diseases and developing stronger, more effective therapeutic strategies are areas that warrant further research.

IL‐1β derived from macrophages and preosteoclasts promotes osteoclast maturation and bone resorption.^[^ ^1^ ^]^ Abnormal IL‐1β production, activation of NLRP3 inflammasomes, and GSDMD‐N terminal cleavage were observed in the early stages of RANKL‐induced osteoclastogenesis, indicating that pyroptosis may serve as a critical mechanism for IL‐1β release and mediating cell interactions.^[^ ^4^ ^]^ The NLRP3 inflammasome, composed of the sensor NLRP3, the adaptor ASC, and the effector caspase‐1, plays a central role in innate immunity and inflammation by mediating pyroptosis.^[^ ^5^ ^]^ ASC connects to NLRP3 through its PYD domain and to caspase‐1 via its CARD domain. The exact mechanism of NLRP3 inflammasome activation is still unclear, but it generally involves two steps: initiation and activation.^[^ ^6^ ^]^ Once activated, caspase‐1 promotes the maturation of IL‐1β and IL‐18, while gasdermin D (GSDMD) facilitates their release by creating pores in the cell membrane. Abnormal activation of NLRP3‐mediated pyroptosis has been linked to the progression of inflammatory bone loss disorders such as osteoporosis, periprosthetic osteolysis, rheumatoid arthritis, psoriatic arthritis, and ankylosing spondylitis. In animal models of sepsis and colitis, certain NLRP3‐targeted inhibitors have shown promising anti‐inflammatory effects and the potential to prevent inflammatory diseases.^[^ ^7^ ^]^ Our previous research demonstrated that inhibiting NF‐κB‐activated pyroptosis reduced abnormal osteoclastogenesis and alleviated osteoporosis and rheumatoid arthritis.^[^ ^8^ ^]^ Clearly, targeting NLRP3 inflammasomes presents a promising therapeutic strategy for treating inflammatory bone loss.^[^ ^9^ ^]^ However, the specific mechanisms by which NLRP3 inflammasomes contribute to these conditions remain to be fully elucidated.

Pinosylvin (PIN) is a natural phenolic compound, named after its isolation from the European red pine (Pinus sylvestris), and exhibits various biological activities, including anti‐inflammatory, antioxidant, and anti‐tumor effects.^[^ ^10^ ^]^ PIN was found to inhibit IL‐1‐ and IL‐17‐induced IL‐6 production in osteoarthritis (OA) chondrocytes and to increase aggrecan level.^[^ ^11^ ^]^ Similarly, in both LPS‐induced M1 macrophage polarization and IL‐4‐induced M2 macrophage polarization, PIN enhances the expression of PPAR‐γ and STAT6, thereby inhibiting the downstream secretion of IL‐1 and TNF‐α.^[^ ^12^ ^]^ In addition, PIN derivatives, 3,5‐dimethoxytrans‐stilbene and 3‐hydroxy‐5‐benzyloxytrans‐stilbene, also significantly inhibited COX‐2 mRNA expression and PGE2 production in LPS‐induced RAW 264.7 cells.^[^ ^13^ ^]^ However, the role and specific mechanisms of PIN in inflammatory bone loss remain unclear. We aim to focus on studying its biological effects on inflammatory bone loss and the underlying mechanisms of its anti‐inflammatory actions.

In this study, we found that both in vivo and in vitro, PIN alleviated abnormal osteoclastogenesis and bone loss under inflammatory conditions by reducing IL‐1β secretion, but not TNF‐α or IL‐6. Additionally, our results showed that PIN inhibited the maturation of IL‐1β and the cleavage of GSDMD by reducing the oligomerization and assembly of the NLRP3 inflammasome. Mechanistically, PIN attenuated NLRP3 inflammasome‐mediated pyroptosis and abnormal osteoclastogenesis by blocking the NEK7/NLRP3 interaction. In conclusion, our study demonstrates that the natural extract PIN exerts anti‐inflammatory and anti‐osteoporotic effects by targeting the NLRP3 inflammasome, broadening its potential as a therapeutic agent.

## 2 Result

### 2.1 PIN Alleviates Abnormal Osteclastogenesis and Inflammatory‐Related Bone Loss by Inhibiting IL‐1β

**Figure 1:**
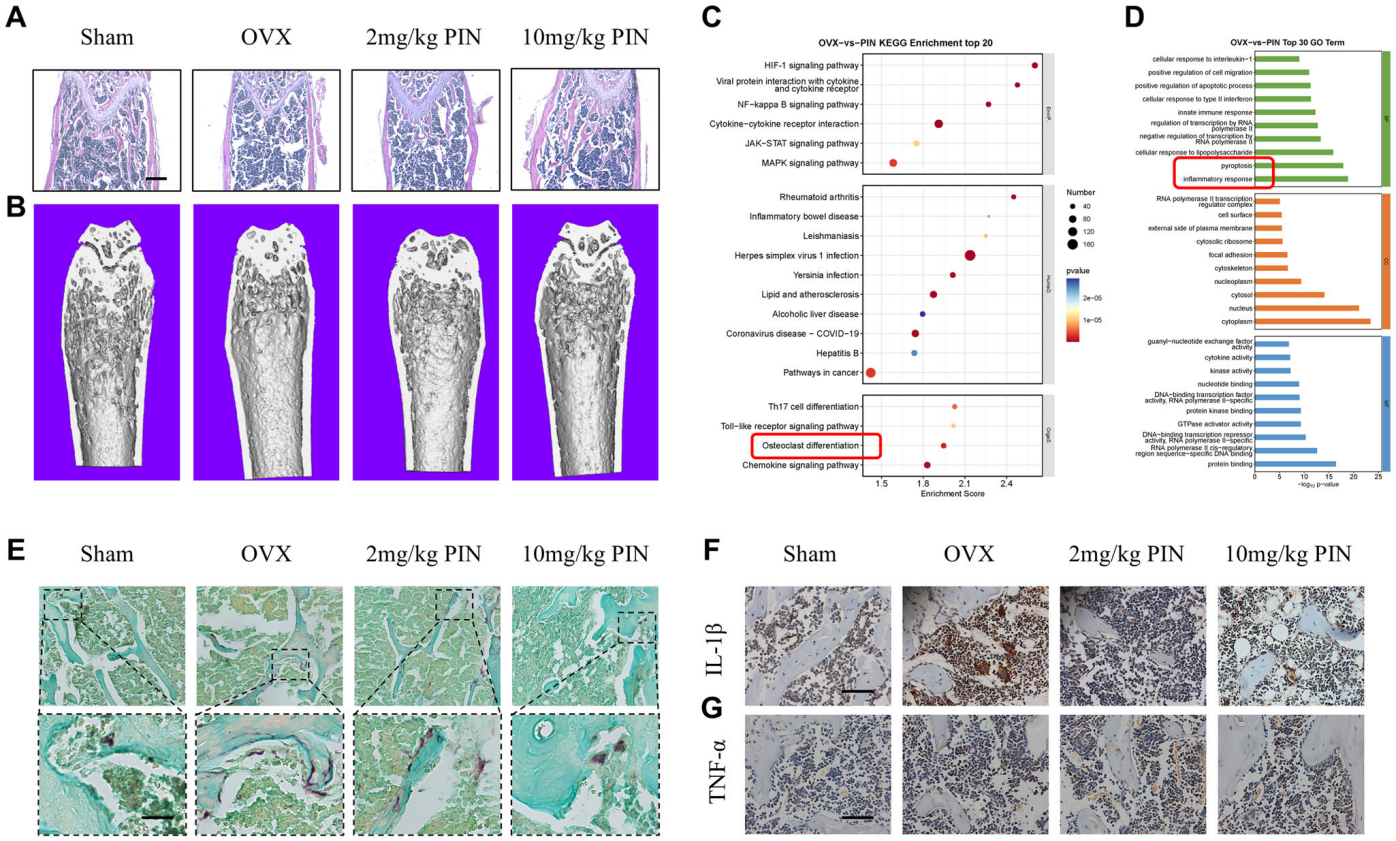
PIN alleviates abnormal osteclastogenesis and inflammatory bone loss in IL‐1β‐dependent manner. A) Representative HE images of femur in each group. n = 7, Scale bar = 200 µm. B) Representative 3D reconstruction (micro‐CT) image of femur in each group. n = 7. C) Top 20 of KEGG enrichment after PIN treatment in OVX mice. D) Top 30 of GO enrichment after PIN treatment in OVX mice. E) Representative TRAP images of femur in each group. Scale bar = 50 µm. n = 7. F,G) Representative staining of IL‐1β and TNF‐α immunohistochemistry from each group. n = 7. Scale bar = 50 µm.

To explore the potential role of PIN in inflammatory‐related bone loss, we established an ovariectomy‐induced osteoporosis (OVX) model, which simulates inflammation and bone loss. First, we observed that PIN alleviated OVX‐induced bone loss in a dose‐dependent manner compared to untreated OVX mice, increased bone mineral density (BMD), bone volume fraction (BV/TV), trabecular number (TB.N), and reduced trabecular separation (TB.Sp) (**Figure** **1**A,B; Figure S1, Supporting Information). Subsequently, we extracted bone marrow cells from OVX mice and high‐dose PIN‐treated mice 8 weeks postoperatively and conducted RNA sequencing (RNA‐seq) assays. The heat map and volcano plot indicate significant differences in gene expression between the two cell groups (Figure S2, Supporting Information). Importantly, KEGG and GO analysis indicated that the differential expression was primarily enriched in biological processes, including pyroptosis, inflammation and osteoclast differentiation (Figure 1C,D). TRAP staining revealed that PIN treatment reduced OVX‐induced abnormal osteoclastogenesis in a dose‐dependent manner (Figure 1E; Figure S3A, Supporting Information). Immunohistochemical staining indicated that PIN alleviated OVX‐induced IL‐1β overload in cancellous bone, but had no effect on OVX‐induced TNF‐α elevation (Figure 1F,G; Figure S3B,C, Supporting Information). In addition, PIN treatment showed no signs of organ damage in OVX mice (Figure S4, Supporting Information). These results suggest that PIN mitigates osteoporosis progression by inhibiting abnormal osteoclastogenesis in inflammatory conditions. In addition, we observed that PIN partially promoted bone formation in vivo and in vitro (Figures S5 and S6, Supporting Information).

To explore the impact of PIN on systemic inflammation, we monitored serum levels of IL‐1β, TNF‐α, and IL‐6 at 2, 4, and 8 weeks post‐OVX and PIN treatment. ELISA results indicated that serum levels of IL‐1β, TNF‐α, and IL‐6 were elevated 2 weeks after OVX. PIN treatment reduced IL‐1β levels starting at 4 weeks but showed no significant effects on serum TNF‐α or IL‐6 levels (Figure S7, Supporting Information). These results suggest that PIN alleviates OVX‐induced systemic inflammation by reducing IL‐1β secretion.

### 2.2 PIN Inhibits Abnormal Osteclastogenesis under Inflammatory Condition in Vitro

**Figure 2:**
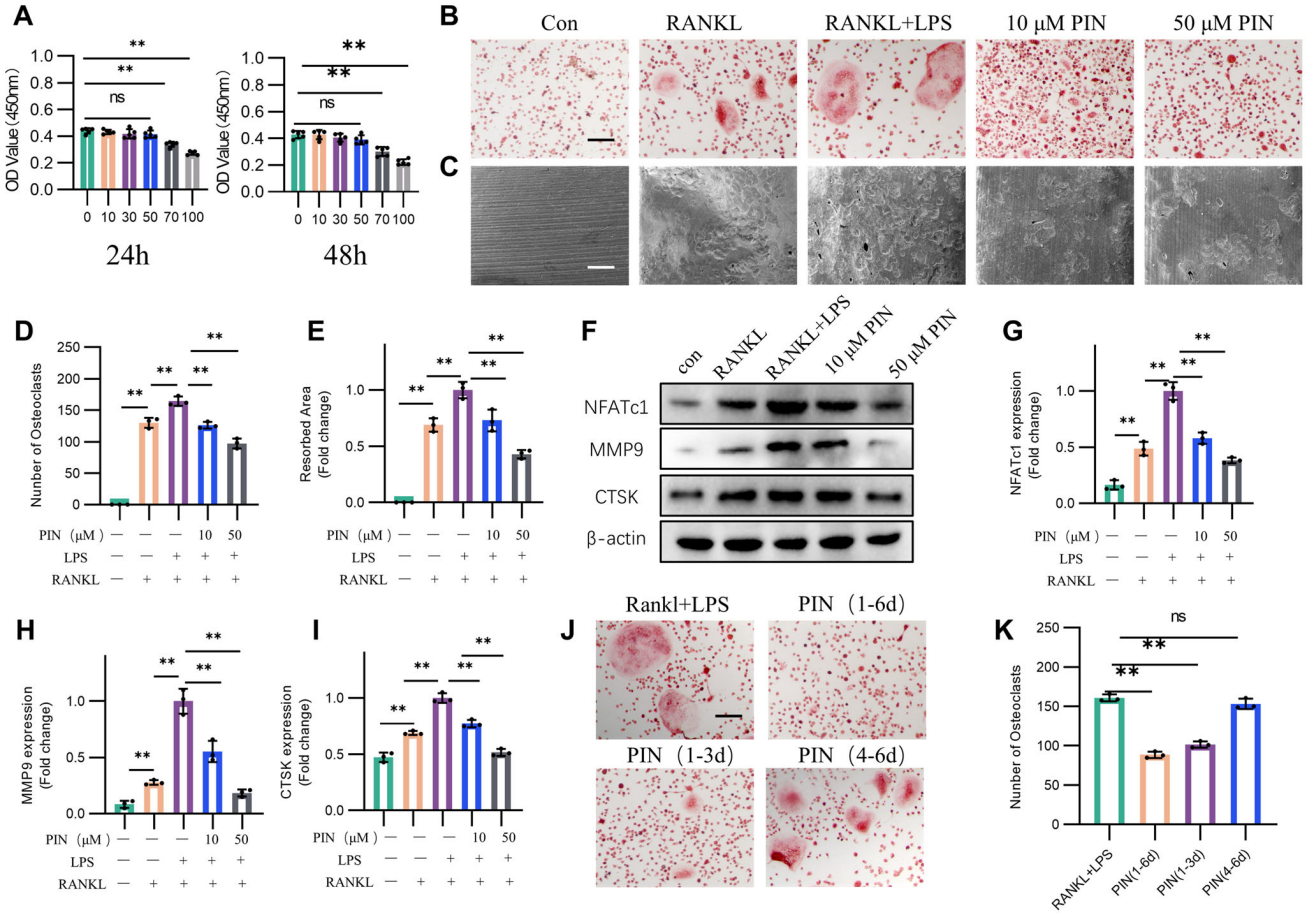
PIN inhibits abnormal osteclastogenesis under inflammatory condition in vitro. A) Cell Counting Kit‐8 (CCK 8) assessed cell viability with 0–100 µM PIN in BMDMs at 24h or 48h. n = 5. B) TRAP staining to evaluate the size of OCs in LPS‐ and RANKL‐induced osteoclastogenesis with PIN treatment. Scale bar = 200 µm. n = 3. C) Bone plate resorption assay to evaluate the bone resorption of OCs in LPS‐ and RANKL‐induced osteoclastogenesis with PIN treatment. Scale bar = 50 µm. n = 3. D) Quantitative analysis of the number of OCs in TRAP staining. n = 3. E) Quantitative analysis of bone resorption in each group. n = 3. F–I) Western blotting images and quantitative analysis of NFATc1, MMP9 and CTSK in LPS‐ and RANKL‐induced osteoclastogenesis with PIN treatment. n = 3. J,K) TRAP images and quantitative analysis during osteoclastogenesis with PIN treatment of different periods. n = 3. **p < 0.01; ns: p >0.05.

Macrophages secreting inflammatory cytokines such as IL‐1β, TNF‐α, and IL‐6 and Osteoclasts affecting bone remodeling share the same precursor cells (bone marrow‐derived macrophages, BMDMs) but differentiate into distinct functional cells based on specific signaling pathways and microenvironmental cues.^[^ ^14^ ^]^ CCK‐8 analysis revealed that treatment with 0–50 µm PIN showed no significant cytotoxicity to BMDMs after 24 or 48 h (**Figure** **2**A). To investigate the effects of PIN on macrophages and OCs, we treated cells with 100 ng mL^−1^ LPS and 50 ng mL^−1^ RANKL to mimic abnormal osteoclastogenesis under inflammatory conditions. TRAP staining and the bone resorption assay showed that treatment with LPS and RANKL significantly increased the size and number of OCs, as well as their bone resorption capacity, compared to RANKL treatment alone. However, PIN, particularly at a concentration of 50 µm, reduced both the size and number of OCs, as well as their bone resorption capacity, under LPS and RANKL stimulation (Figure 2B–E). Similar results were obtained by extracting whole‐cell proteins from each group and detecting the osteoclast markers NFATc1, MMP9, and CTSK (Figure 2F–I). Subsequently, we treated the LPS and RANKL‐induced inflammatory osteoclastogenesis with 50 µm PIN on days 1–6, 1–3, and 4–6. TRAP staining revealed that PIN significantly reduced osteoclastogenesis when administered throughout the entire period or during days 1–3. However, treatment on days 4–6 had no noticeable effect, suggesting that PIN's inhibitory impact on abnormal osteoclastogenesis under inflammatory conditions primarily occurs during the early stages of induction (Figure 2J, K).

### 2.3 PIN Inhibits Canonical Pyroptosis During Osteoclastogenesis Under Inflammation

**Figure 3:**
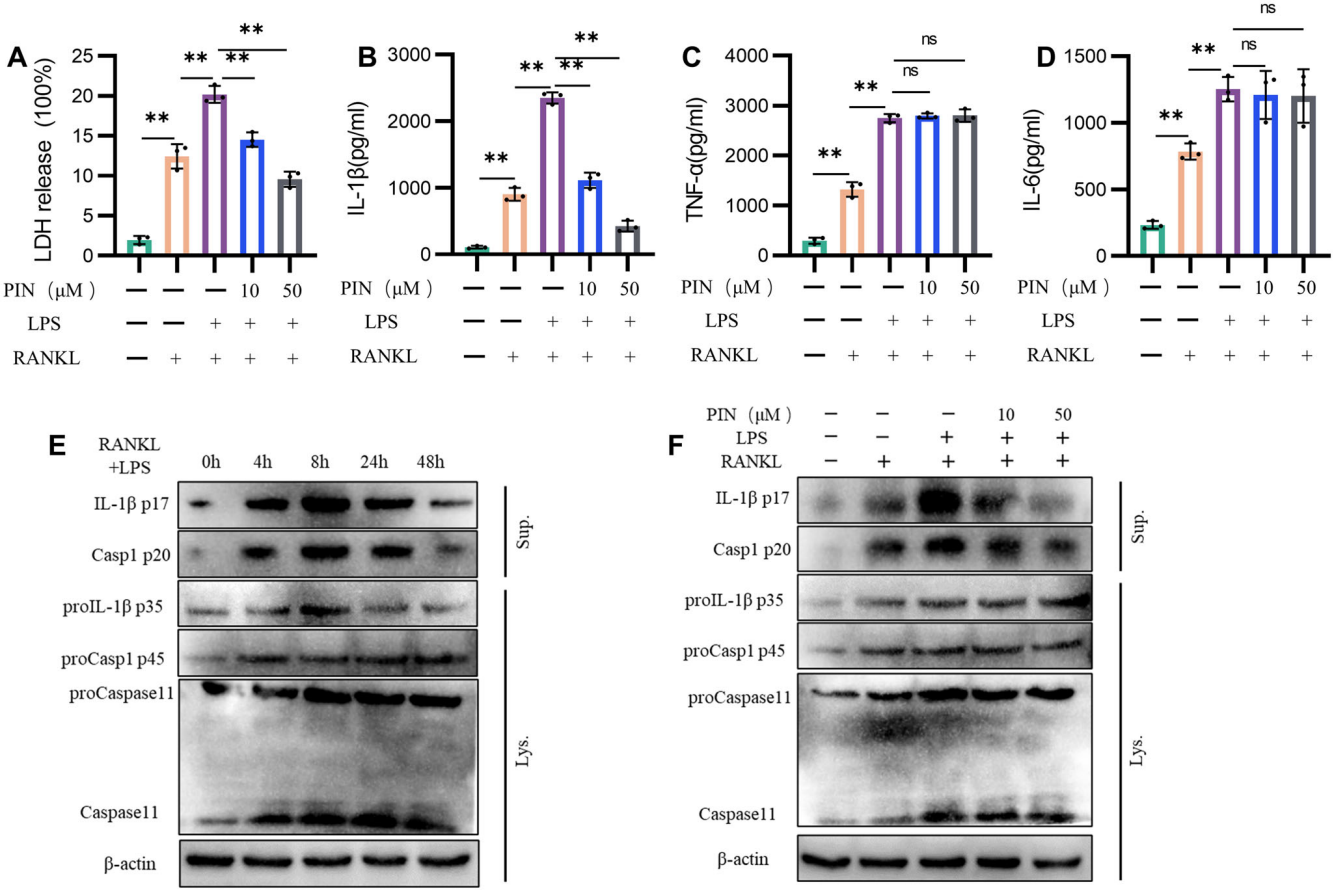
PIN inhibits canonical pyroptosis during inflammatory osteoclastogenesis. A–D) LDH, IL‐1β, TNF‐α and IL‐6 were detected by supernatant ELISA after LPS and RANKL treated BMDMS for 48 h. n = 3. E) BMDMs were stimulated with LPS and RANKL for 0, 4, 8, 24, 48 h. Cell lysates and supernatants were analysed by immunoblotting. n = 3. F) BMDMs were treated with PIN for 1h, and then stimulated with LPS and RANKL for 8 h. Cell lysates and supernatants were analyzed by immunoblotting. n = 3. **p < 0.01; ns: p >0.05.

Subsequently, we collected cell supernatants at 24 h for ELISA analysis to investigate the effect of PIN on the release of LDH, IL‐1β, TNF‐α and IL‐6. TNF‐α and IL‐6 are classical secreted proteins, with their release relying on the conventional endoplasmic reticulum‐Golgi secretion pathway, whereas LDH and IL‐1β release primarily depend on NLRP3 inflammasome‐related pyroptosis.^[^ ^15^ ^]^ The results demonstrated that RANKL treatment triggered the release of LDH, IL‐1β, TNF‐α, and IL‐6, indicating that RANKL induced pyroptosis and activated the endoplasmic reticulum‐Golgi secretion pathway (**Figure** **3**A–D). Subsequently, we examined the specific effects of LPS and RANKL on IL‐1β production using Western blot analysis. The results showed that RANKL alone increased the expression of Pro‐IL‐1β and promoted its cleavage into mature IL‐1β. In contrast, LPS stimulation led to an upregulation of Pro‐IL‐1β without promoting its maturation or release. Moreover, the combined stimulation with LPS and RANKL resulted in a significantly higher level of mature IL‐1β compared to RANKL alone, suggesting a synergistic effect in facilitating IL‐1β maturation and release (Figure S8A, Supporting Information). Notably, PIN treatment significantly reduced the release of LDH and IL‐1β but not TNF‐α and IL‐6, suggesting that PIN effectively inhibits LPS and RANKL‐induced pyroptosis (Figure 3A–D). Similarly, PIN also demonstrated therapeutic efficacy in pyroptosis induced by LPS and monosodium urate (MSU) (Figure S8B, Supporting Information).

To determine whether canonical and non‐canonical pyroptosis activated during LPS and RANKL treatment, we examined the effects of PIN on the cleavage of caspase‐1, caspase‐11, and the secretion of IL‐1β. LPS and RANKL treatment promoted caspase‐1 and caspase‐11 cleavage, as well as IL‐1β secretion, with the most pronounced effects observed 8 h post‐treatment (Figure 3E). We subsequently treated PIN‐treated BMDMs with LPS and RANKL for 8 h. RANKL treatment induced the production and cleavage of Pro‐IL‐1β, Pro‐caspase‐1, and Pro‐caspase‐11, with the effect being significantly enhanced when combined with LPS. Importantly, PIN exhibited dose‐dependent inhibition of caspase‐1 cleavage and IL‐1β secretion in response to LPS and RANKL treatment, but had no effect on caspase‐11 cleavage (Figure 3F). These results suggest that PIN reduces the release of inflammatory factors by specifically inhibiting canonical pyroptosis.

### 2.4 PIN Blocks ASC Assembly and GSDMD Cleavage Stimulated by LPS and RANKL in Macrophages

**Figure 4:**
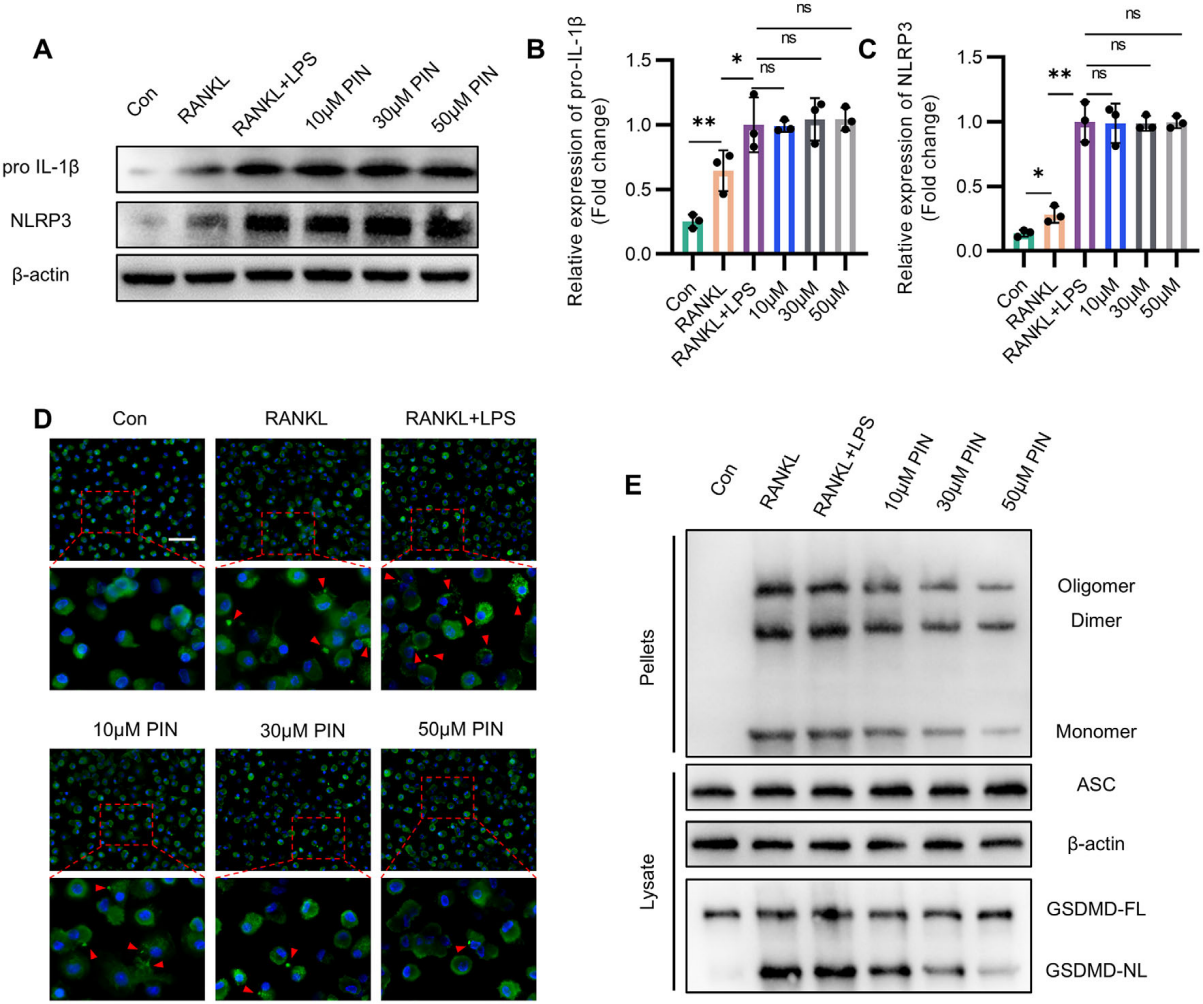
PIN blocks ASC assembly and GSDMD cleavage stimulated by LPS and RANKL in macrophages. A–C) Western blotting images and quantitative analysis of pro‐IL‐1β and NLRP3 in LPS‐ and RANKL‐induced osteoclastogenesis with PIN treatment. n = 3. D) Representative immunofluorescence images of ASC speck formation in BMDMs stimulated with LPS and RANKL in the presence of PIN. Scale bar = 200 µm. n = 3. E) Analysis of ASC oligomerization in cross‐linked cytosolic pellets and cleavage of GSDMD by immunoblotting. n = 3. *p < 0.05;**p < 0.01; ns: p >0.05.

Priming is the first step in NLRP3 inflammasome formation, typically triggered by cell surface receptors (e.g., TLRs) that recognize PAMPs or DAMPs. This activation stimulates the downstream NF‐κB pathway, leading to increased expression of NLRP3, Pro‐IL‐1β, and Pro‐IL‐18. Our study found that PIN, at various concentrations, did not alter LPS and RANKL‐induced overexpression of priming substrates Pro‐IL‐1β and NLRP3, suggesting that PIN's inhibitory effect on canonical pyroptosis does not take place during the priming step (**Figure** **4**A–C). To determine whether PIN influences the activation step of the NLRP3 inflammasome during inflammatory osteoclastogenesis, we assessed ASC nucleation‐induced oligomerization or polymerization, as well as GSDMD cleavage. PIN dose‐dependently reduced the formation of ASC specks that polymerized and assembled by ASC under LPS and RANKL stimulation (Figure 4D; Figure S9, Supporting Information). Similarly, ASC oligomerization and GSDMD cleavage were significantly reduced with PIN intervention (Figure 4E). In addition, the inhibitory effect of PIN on ASC oligomerization was also observed in pyroptosis induced by LPS and MSU (Figure S10, Supporting Information). These results suggest that PIN inhibits canonical pyroptosis in early stage of osteoclastogenesis by blocking ASC oligomerization, speck formation, and assembly.

### 2.5 The Inhibitory Effect of PIN on NLRP3 Inflammasomes is Independent of Mitochondrial Oxidative Stress, Potassium Efflux, or Calcium Influx

**Figure 5:**
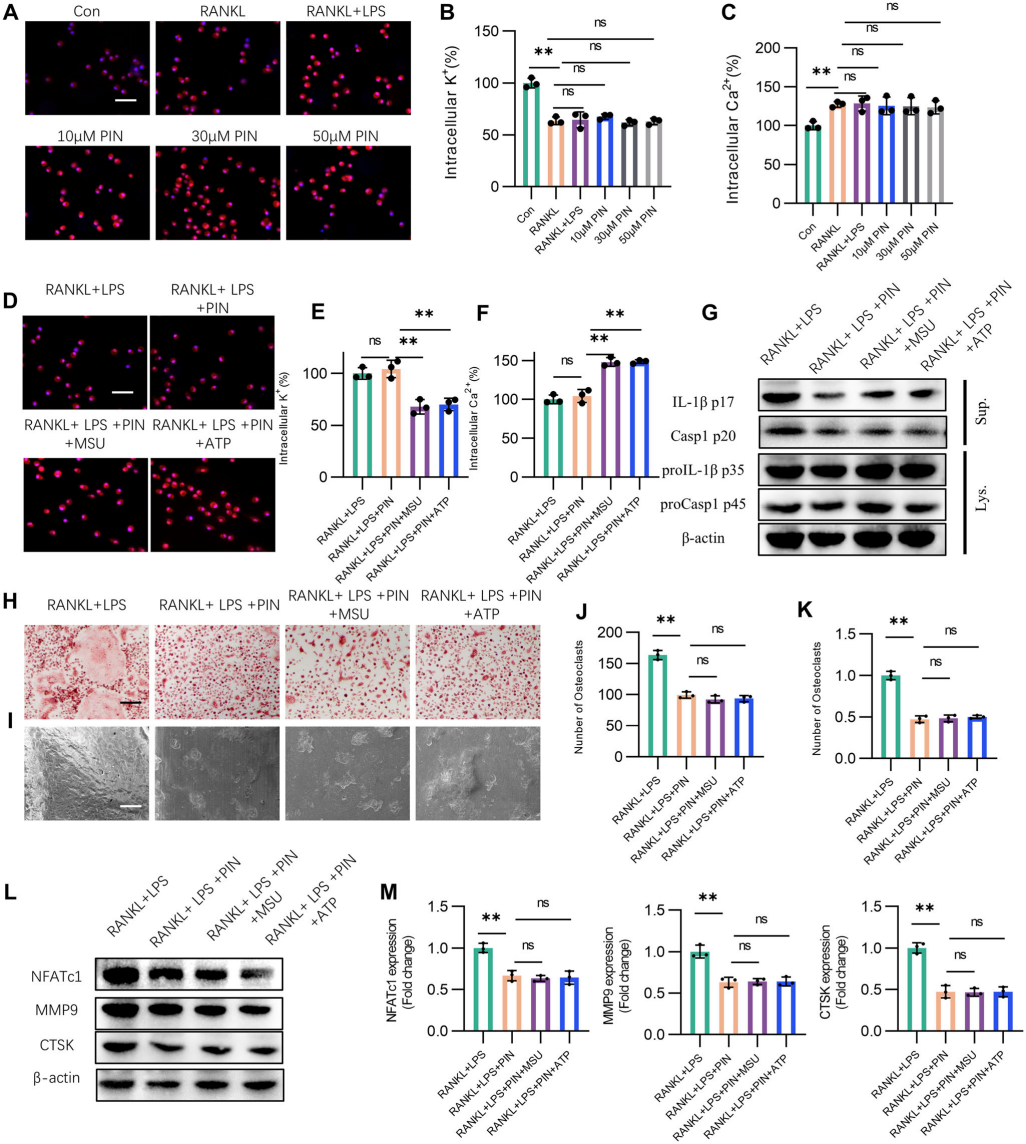
The inhibitory effect of PIN on NLRP3 inflammasomes is independent of mitochondrial oxidative stress, potassium efflux, or calcium influx. A) Analysis of mtROS in LPS‐ and RANKL‐induced osteoclastogenesis with PIN treatment. n = 3. B, C) Analysis of cellular K^+^ and Ca^2+^ in LPS‐ and RANKL‐induced osteoclastogenesis with PIN treatment by ICP‐MS. n = 3. D–F) Analysis of mtROS, cellular K^+^ and Ca^2+^ in PIN pre‐treated BMDMs that was stimulated with LPS, RANKL and MUS or ATP. n = 3. G) Cleavage of IL‐1β and Caspase‐1 in PIN pre‐treated BMDMs that was stimulated with LPS, RANKL and MUS or ATP. n = 3. H–K) Analysis of TRAP staining and bone resorption in PIN pre‐treated BMDMs that was stimulated with LPS, RANKL and MUS or ATP for 6 days. n = 3. L,M) Western blotting images and quantitative analysis of NFATc1, MMP9 and CTSK i in PIN pre‐treated BMDMs that were stimulated with LPS, RANKL and MUS or ATP for 6 days. n = 3. **p < 0.01; ns: p >0.05.

Recent studies have identified mitochondrial oxidative stress, potassium efflux, and calcium influx as the three major factors that initiate NLRP3 inflammasome activation.^[^ ^16^ ^]^ Our findings indicate that PIN treatment did not influence enhanced mitochondrial oxidative stress, potassium efflux, or calcium influx induced by the combined treatment of LPS and RANKL in BMDMs (**Figure** **5**A–C; Figure S11A, Supporting Information). To further clarify the specific targets of PIN in alleviating pyroptosis, we treated BMDMs with the NLRP3 inflammasome activators MSU and adenosine triphosphate (ATP) along with LPS, RANKL and PIN for 8 h. In the presence of PIN, MSU and ATP further amplified LPS and RANKL‐induced mitochondrial oxidative stress, potassium efflux, and calcium influx (Figure 5D–F; Figure S11B, Supporting Information). Interestingly, neither MSU nor ATP enhanced the cleavage or release of caspase‐1 and IL‐1β under PIN stimulation (Figure 5G). Furthermore, extending the intervention time to 6 days, MSU and ATP still failed to increase osteoclast numbers, bone resorption capability, or the expression of NFATc1, MMP9 and CTSK involved in bone resorption in the presence of PIN (Figure 5H–M). These results suggest that PIN's inhibition of pyroptosis is not dependent on mitochondrial oxidative stress, potassium efflux, or calcium influx, but rather occurs downstream of these events.

### 2.6 PIN Blocks NEK7/NLRP3 Interaction by 3,5‐Dihydroxy

**Figure 6:**
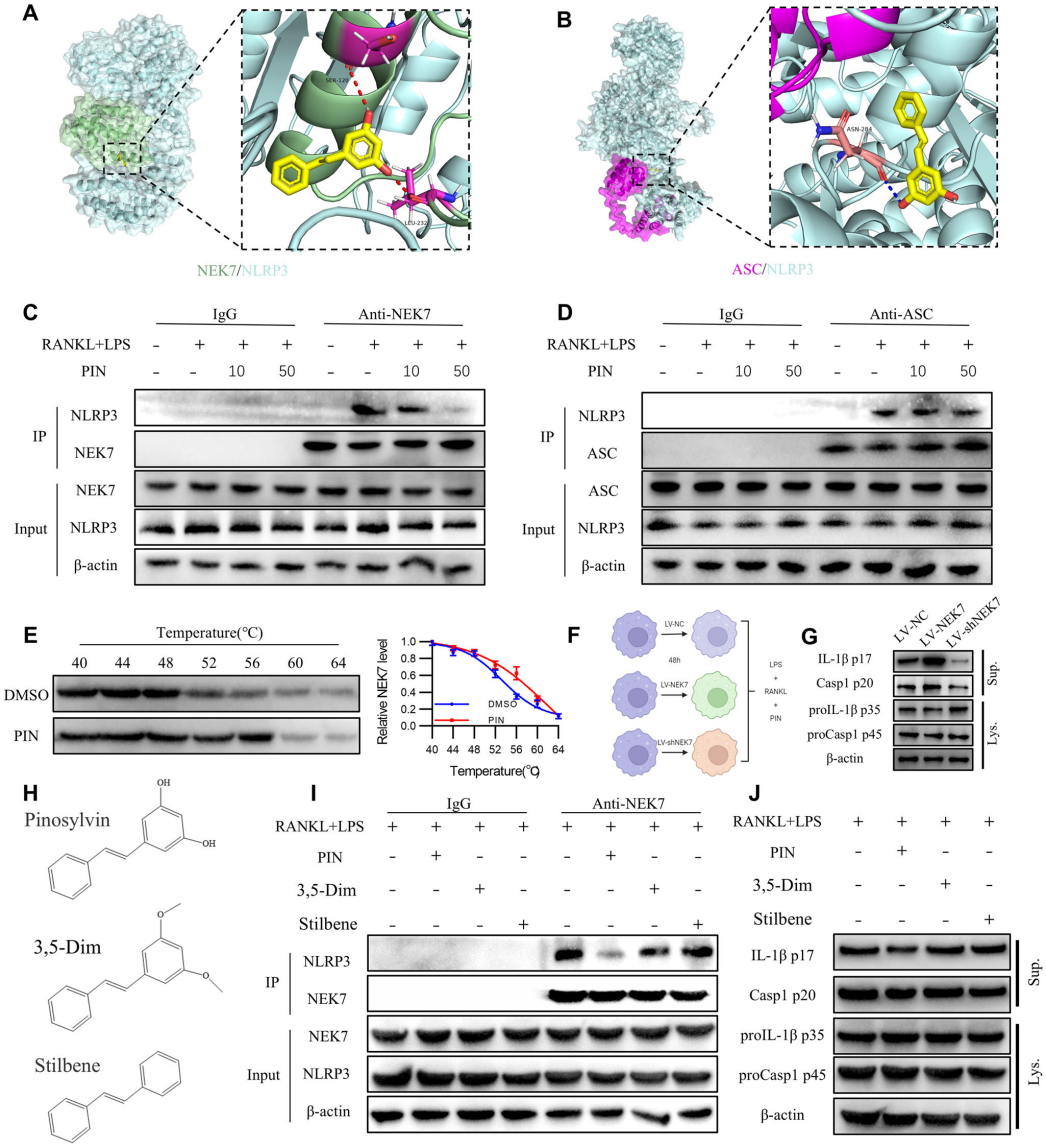
PIN blocks NEK7/NLRP3 interaction by 3,5‐Dihydroxy. A,B) Molecular docking of PIN to NEK7/NLRP3 complex and ASC/NLRP3 complex. C,D) Co‐IP analysis of NEK7/NLRP3 and ASC/NLRP3 in PIN pre‐treated BMDMs that was stimulated with LPS and RANKL. IgG was used as an internal control, and cell lysates are shown as inputs. n = 3. E) DARTS analysis of PIN‐treated BMDMs. n = 3. F) Diagram of osteoclastogenesis under inflammatory conditions in LV‐NEK7 or LV‐shNEK7 BMDMs. G) Cleavage of IL‐1β and Caspase‐1 in LV‐NEK7 and LV‐shNEK7BMDMs stimulated with LPS and RANKL. n = 3. H) Structure of pinosyvin, 3,5‐dimethoxystilbene and stilbene. I) Co‐IP analysis of NEK7/NLRP3 and ASC/NLRP3 in 3,5‐Dim or stilbene pre‐treated BMDMs that were stimulated with LPS and RANKL. n = 3. J) Cleavage of IL‐1β and Caspase‐1 in 3,5‐Dim or stilbene pre‐treated BMDMs. n = 3.

We found that PIN did not affect the priming of pyroptosis or signaling events such as mitochondrial oxidative stress, potassium efflux, and calcium influx in inflammatory osteoclastogenesis. However, it inhibited ASC assembly, suggesting that PIN may interfere with the formation of NLRP3 inflammasomes. The interaction between NEK7 and NLRP3, along with the recruitment of NLRP3 by ASC, are two essential steps for the subsequent formation of NLRP3 inflammasomes.^[^ ^17^ ^]^ To evaluate the affinity of PIN for the NEK7/NLRP3 and ASC/NLRP3 complexes, we conducted molecular docking analysis. The results predicted that PIN occupies the hydrophobic pockets of both complexes and binds to them via hydrogen bonding. Molecular docking analysis predicted PIN interacts with SER‐120 and LEU‐232 of NEK7 through its 3,5‐dihydroxy groups in the NEK7/NLRP3 complex; PIN binds to ASN‐284 of NLRP3 via its 3‐hydroxy group in the ASC/NLRP3 complex (**Figure** **6**A,B). We then assessed the effect of PIN on NLRP3's ability to bind either NEK7 or ASC. Co‐IP results revealed that PIN dose‐dependently reduced the interaction between NEK7 and NLRP3, but had no effect on the interaction between ASC and NLRP3 (Figure 6C,D). This suggests that PIN inhibits downstream NLRP3 inflammation activation by reducing the NEK7/NLRP3 interaction.

We next performed cellular thermal shift assay (CETSA) and nano Differential Scanning Fluorimetry (nanoDSF), which measure protein thermal stability. CESTA revealed higher levels of NEK7 protein in the PIN‐treated group compared to the DMSO group at temperatures between 52 and 56 °C (Figure 6E). The nanoDSF results also suggest that PIN increases the thermal stability of NEK7, as indicated by an elevated melting temperature (Tm) (Figure S12, Supporting Information). To further investigate whether PIN exerts its anti‐pyroptosis effect through NEK7, we employed lentiviral overexpression (LV‐NEK7) and knockdown (LV‐shNEK7) of NEK7 (Figure 6F). After 8 h treatment, LV‐NEK7, but not LV‐shNEK7 reversed the PIN‐induced inhibition of IL‐1β and caspase‐1 cleavage and release (Figure 6G). Notably, after 6 days, LV‐NEK7, but not LV‐shNEK7 enhanced osteoclastogenesis, bone resorption activity, and the expression of NFATc1, MMP9 and CTSK, even in the presence of PIN (Figure S13, Supporting Information). These results suggest that PIN inhibits subsequent pyroptosis and osteoclastogenesis by binding to NEK7 to reduce the formation of the NEK7/NLRP3 complex.

PIN, also known as 3,5‐dihydroxystilbene, is believed to exert its pharmacological effects through its 3,5‐dihydroxy groups, as suggested by molecular docking. To clarify the role of these hydroxyl groups, we substituted them with methoxy groups to create 3,5‐dimethoxystilbene (3,5‐Dim), and we removed them entirely to form stilbene (Figure 6H). Unlike PIN, neither 3,5‐Dim nor stilbene reduced the LPS and RANKL‐induced binding of NEK7 to NLRP3 (Figure 6I). Similarly, 3,5‐Dim and stilbene did not reduce LPS and RANKL‐induced IL‐1β or caspase‐1 cleavage and release (Figure 6J). Over a 6‐day intervention, 3,5‐Dim and stilbene also failed to attenuate the aberrant osteoclastogenesis, enhanced bone resorption capacity, and upregulation of NFATc1, MMP9, and CTSK, as PIN did (Figure S14, Supporting Information).

In summary, PIN binds to NEK7 via its 3,5‐dihydroxy groups, blocking the NEK7/NLRP3 interaction and inhibiting downstream activation of NLRP3 inflammasomes.

### 2.7 PIN Alleviates the Process of Osteolysis and Sepsis

**Figure 7:**
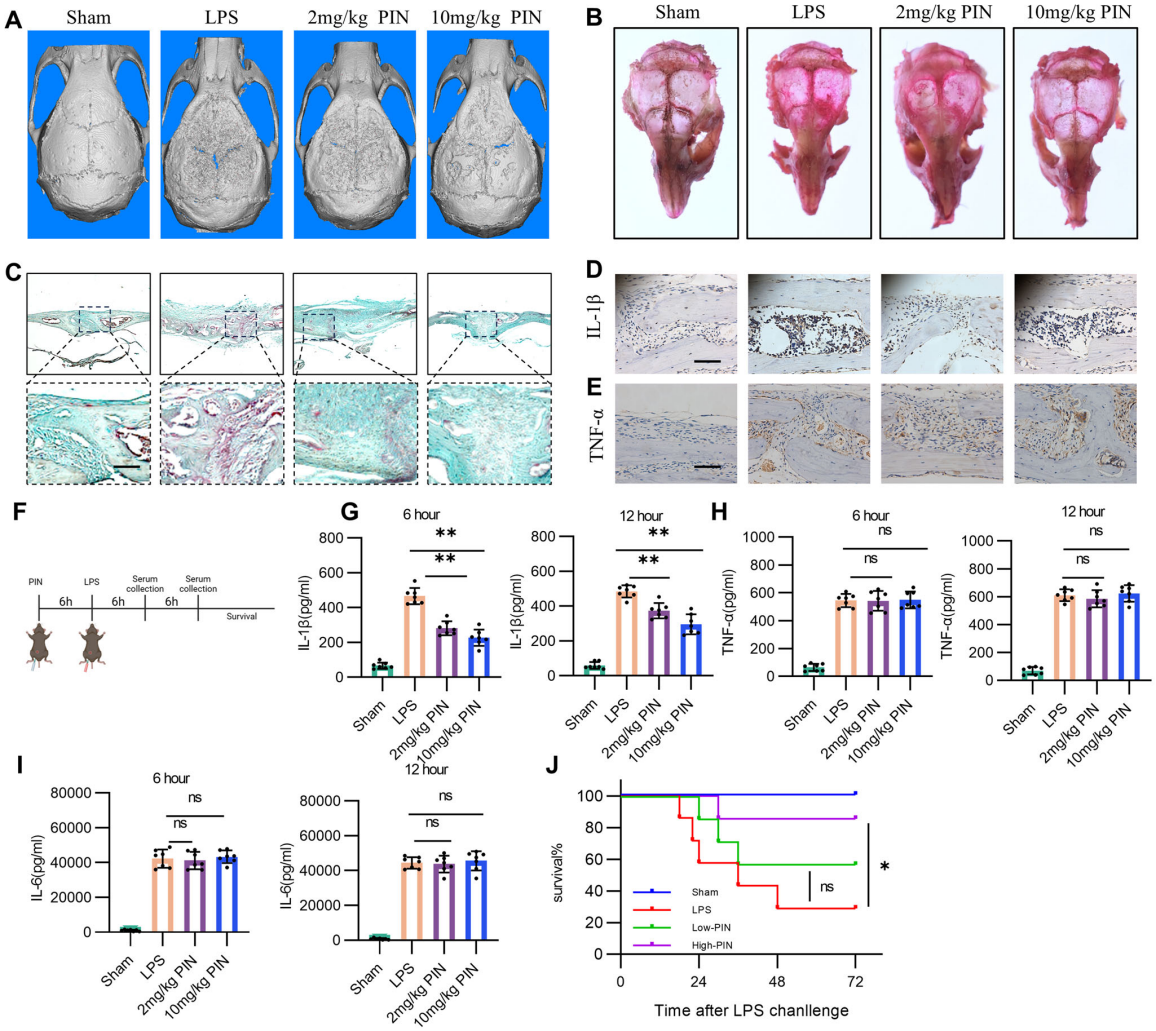
PIN alleviates the process of osteolysis and sepsis. A) Representative 3D reconstruction (micro‐CT) image of skull in each group. n = 7. B) TRAP staining of the entire skull in each group. n = 7. C) TRAP staining of the skull in coronal section. n = 7. D,E) Representative staining of IL‐1β and TNF‐α immunohistochemistry from each group. Scale bar = 50 µm. n = 7. F) LPS‐induced sepsis. G–I) Serum IL‐1β, TNF‐α and IL‐6 in mice were detected by ELISA after LPS treatment for 6 h and 12 h. n = 7. J) Survival of PIN‐treated mice. n = 7. *p < 0.05; **p < 0.01; ns: p >0.05.

In addition to osteoporosis, osteolysis is another inflammatory bone loss disease associated with pyroptosis, which is sensitive to LPS challenge.^[^ ^18^ ^]^ C57BL/6 mice were administered a single subcutaneous cranial injection of 25 mg kg^−1^ LPS to induce cranial osteolytic destruction and bone loss, followed by intraperitoneal injections of 2 and 10 mg kg^−1^ PIN. As expected, micro‐CT images revealed that PIN‐treated mice had a smoother and more intact skull structure compared to LPS‐treated mice (**Figure** **7**A). Similarly, bone parameter analyses showed that in LPS‐induced osteolysis, PIN increased BMD and BV/TV, while decreasing BS/BV and porosity (Figure S15A, Supporting Information). To assess the effect of PIN on osteoclastogenesis in the osteolysis model, we performed TRAP staining on whole skulls and coronal sections. The results indicated that PIN alleviated LPS‐induced abnormal osteoclastogenesis in a concentration‐dependent manner (Figure 7B,C; Figure S15B, Supporting Information). Next, we evaluated the effects of PIN on IL‐1β and TNF‐α levels in the osteolysis model. Consistent with findings in OVX mice, PIN treatment reduced the number of IL‐1β‐positive cells but not TNF‐α‐positive cells on the surface of bone tissue (Figure 7D,E; Figure S15C,D, Supporting Information). These data show that PIN have a protective effect on LPS‐induced osteolysis.

To further validate whether PIN is the therapeutic target of pyroptosis induced disease, LPS mediated sepsis was utilized. Mice pretreated with PIN via intraperitoneal injection 6 h prior were challenged with 25 mg kg^−1^ LPS intraperitoneally. Serum levels of IL‐1β, TNF‐α, and IL‐6 were measured at 6 and 12 h after the LPS challenge, and survival was monitored for 72 h (Figure 7F). Notably, compared to LPS‐induced septic mice, PIN‐treated mice showed lower serum IL‐1β levels at 6 or 12 h, while no significant differences were observed in TNF‐α and IL‐6 (Figure 7G–I). Mice treated with a high dose of PIN began to die at 30 h after the LPS challenge, compared to 18 h in septic mice. Moreover, high PIN‐treated mice exhibited a significantly higher survival rate at 72 h compared to the septic mice (Figure 7J). Thus, these data suggested PIN as a promising target for therapy of pyroptosis‐mediated disease like sepsis.

## 3 Discussion

NLRP3 inflammasome activation induces IL‐1β maturation in response to various infectious and aseptic factors, playing a crucial role in the pathology of inflammatory bone loss.^[^ ^8^, ^19^ ^]^ Targeting NLRP3 inflammasome activation is therefore a key strategy for treating inflammatory bone loss. In this study, we found that PIN effectively blocked NEK7/NLRP3 binding, inhibited the activation of NLRP3 inflammasomes and subsequent pyroptosis, and reduced abnormal osteoclastogenesis and bone loss. These findings offer new hope for the treatment of NLRP3‐driven inflammatory bone loss.

Inflammatory bone loss is primarily characterized by the enrichment and release of IL‐1β, IL‐6, TNF‐α, and abnormal osteoclastogenesis. However, inflammatory bone loss can also result from infection, monstrosity, biological factor defects, and immune system involvement, ultimately leading to abnormal osteoclastogenesis and bone loss. Cellular models alone cannot fully replicate the pathological process of inflammatory bone loss in vivo. RANKL, which binds to the RANK receptor on the surface of osteoclast precursor cells and induces osteoclast differentiation, is the most potent and effective biological factor known to induce osteoclastogenesis and is widely used in in vitro studies.^[^ ^20^ ^]^ However, it does not account for the complex and variable immune environment present in inflammatory bone loss in vivo. Our study found that the combined intervention of LPS and RANKL intensified pyroptosis, the release of inflammatory factors, and the overproduction of osteoclasts in BMDMs compared to RANKL treatment alone, which appears to better mimic the mixed immune microenvironment found in living organisms.

Previous studies have shown that PIN has pro‐apoptotic activity, with 100 µm PIN promoting AMPKα phosphorylation, leading to leukemia cell death.^[^ ^21^ ^]^ In contrast, our study found that 10 or 50 µm PIN could alleviate LPS and RANKL‐induced cell death and IL‐1β release in BMDMs, suggesting that the biological effects of PIN are concentration‐dependent. Additionally, 100 µm PIN has been shown to reduce M1 polarization and the production of TNF‐α and IL‐1β in human U937 macrophages, as well as decrease IL‐6 expression in human chondrocytes by inhibiting the NF‐κB signaling pathway.^[^ ^11^, ^12^ ^]^ Our study found that 10 or 50 µm PIN did not affect the expression of NF‐κB pathway products such as Pro‐IL‐1β and NLRP3, but did reduce the assembly and oligomerization of NLRP3 inflammasomes. This suggests that PIN has a dose‐dependent effect on inflammatory factors. More importantly, these findings indicate that PIN is particularly sensitive to NLRP3 inflammasome activation, making it potentially more effective in treating NLRP3 inflammasome‐dependent diseases.

Our findings suggest that the mechanism by which PIN reduces abnormal osteoclastogenesis under inflammatory conditions lies in its inhibition of NLRP3 inflammasome activation, rather than the priming step. PIN does not affect potassium efflux, calcium efflux, or mitochondrial oxidative stress, which are key initiators of the activation step of NLRP3 inflammasomes. Notably, potassium efflux, driven by altered ATPase activity, plays a role in the metabolic processes of fats, proteins, sugars, nucleic acids, and nucleotides. Various compounds, such as 2,4‐dinitrobenzenesulfonic acid, 3,4‐methylenedioxy‐β‐nitrostyrene, and Bay 11–7082, have been shown to target ATPase for the treatment of NLRP3‐dependent diseases, but the biosafety of these compounds has not yet been systematically evaluated.^[^ ^22^ ^]^ In the presence of PIN, the intensification of potassium efflux, calcium efflux, or mitochondrial oxidative stress did not promote pyroptosis or osteoclastogenesis. This suggests that PIN's specific targets lie downstream of these processes, indicating that its systemic risk may be lower than that of the aforementioned ATPase‐targeting agents.

NEK7, acting downstream of potassium efflux, licenses the assembly and activation of the NLRP3 inflammasome.^[^ ^17^, ^23^ ^]^ Ubiquitination of NEK7 has been shown to attenuate osteoclast overactivation and the progression of periodontitis.^[^ ^24^ ^]^ Helenine reduced LPS‐induced lethal sepsis and MSU‐induced peritonitis by blocking the NEK7/NLRP3 interaction, without affecting the assembly of other inflammasomes, such as NLRC4 and AIM2.^[^ ^25^ ^]^ Berberine also inhibited caspase‐1 and IL‐1β cleavage by directly targeting NEK7, forming a hydrogen bond between the 2,3‐methylenedioxy group and the R121 residue of NEK7.^[^ ^26^ ^]^ These findings suggest that targeting NEK7 is an effective strategy for treating NLRP3‐dependent diseases. Interestingly, PIN's 3,5‐dihydroxyl groups formed hydrogen bonds with SER‐120 and LEU‐232 of NEK7, leading to the dissociation of the NEK7/NLRP3 complex. Overexpression of NEK7 rescued downstream pyroptosis and promoted osteoclastogenesis at a fixed dose of PIN, suggesting that PIN's blocking effect on NLRP3 inflammasomes is competitive rather than noncompetitive. Hydroxyl groups are the primary functional groups that enable small molecule compounds to exert their biological effects. When the 3,5‐dihydroxyl groups of PIN were removed or replaced, its inhibitory effect on pyroptosis and osteoclastogenesis disappeared, indicating that the therapeutic effect of PIN is primarily mediated by these hydroxyl groups. Now that the effector functional groups of PIN have been identified, further in‐depth studies are needed to modify PIN to improve its poor aqueous solubility and reduce the risk of high‐dose biotoxicity. Previous studies have shown that natural Chinese herbal medicines and compounds, such as Curculigo orchioides Gaertn, Psoralea corylifolia (L.) Medik, and Eucommia ulmoides Oliv, can help maintain bone remodeling homeostasis and mitigate the progression of osteoporosis.^[^ ^27^ ^]^ In our study, we identified a specific mechanism through which PIN alleviates osteoclast activation, and we also demonstrated its therapeutic potential in promoting osteogenic differentiation and mineralization. However, the detailed molecular mechanisms underlying these effects remain unclear and warrant further investigation. These efforts are essential to advancing the clinical application of PIN and its derivatives.

In both estrogen deficiency‐ and LPS‐induced inflammatory bone loss models, PIN effectively reduced IL‐1β levels and alleviated abnormal osteoclast activation. Additionally, high doses of PIN improved the survival rate in LPS‐induced septic mice, suggesting that PIN can target pyroptosis‐related diseases beyond inflammatory bone loss. Moreover, PIN was shown to be safe, with no observed organ damage in mice, positioning it as a promising candidate for clinical inflammation therapy. Other NLRP3 inhibitors, such as OLT1177, have already undergone phase II clinical trials for acute gouty arthritis, and oridonin, the main active compound in the over‐the‐counter herb Rabdosia rubescens, has been indirectly used in clinical treatments.^[^ ^28^ ^]^ However, the clinical application of PIN has not advanced as quickly as anticipated. In conclusion, PIN targets NEK7/NLRP3 interactions, reduces macrophage pyroptosis, alleviates abnormal osteoclastogenesis, and offers a potential treatment for inflammatory bone loss.

## 4 Experimental Section

### Mice

Eight‐week‐old C57BL/6 mice were obtained from the Laboratory Animal Center of Soochow University. The researchers conducted blinded animal experiments, randomly assigning the mice into groups and housing them in specific sterile facilities under controlled conditions (12 h light/12 h dark; 26 ± 2 °C). All animal experiments were approved by the Laboratory Animal Welfare and Ethics Committee of Soochow University.

### Reagents

Pinosylvin (P168709), LPS (L118716), MSU (M276559), 3,5‐dimethoxystilbene (D405584), and Stilbebe (S107167) were purchased from Aladdin. M‐CSF and RANKL were purchased from R&D Systems. Fetal bovine serum (209 111) was purchased from NEST Biotechnology. ATP were purchased from MedChemExpress. Anti‐ GSDMD (1:1000, R40136), and HRP‐Polyclonal Goat Anti‐Rabbit (H+L) IgG (1:4000, KT‐SA‐HP‐02) were purchased from Ketu Biotech. Anti‐β‐actin (1:1000, Ac006) and anti‐ASC (1:1000, A24165) were purchased from ABclonal. Anti‐IL‐1β (1:200, ab283818), anti‐TNF‐α (1:200, ab307164), anti‐NFATc1 (1:1000, ab251916), anti‐MMP9 (1:1000, ab283594), anti‐CTSK (1:1000, ab300569), anti‐caspase‐1 (1:1000, ab207802), anti‐caspase‐11 (1:1000, ab246496), anti‐NEK7 (1:30, ab133514), anti‐NLRP3 (1:30, ab263899), anti‐ASC (1:30, ab283684), anti‐Runx2 (1:1000, ab192256), anti‐Osterix (1:1000, ab209484), and anti‐OCN (1:1000, ab133612)was from abcam.

### OVX‐Induced OP

Eight‐week‐old female C57BL/6 mice underwent ovariectomy and received weekly intraperitoneal injections of 2 or 10 mg kg^−1^ PIN (dissolved in saline containing 5% DMSO) one day after surgery (n = 7). Blood samples were collected at 2, 4, and 8 weeks after OVX, and serum levels of IL‐1β, TNF‐α, and IL‐6 were measured. Eight weeks after surgery, mouse femurs were harvested for micro‐CT analysis and histological staining. Additionally, bone marrow cells from the OVX group and the 10 mg kg^−1^ PIN group were collected for RNA sequencing.

### LPS‐Induced Osteolysis

Eight‐week‐old male C57BL/6 mice received cranial subcutaneous injections of LPS (20 mg kg^−1^) and weekly intraperitoneal injections of 2 or 10 mg kg^−1^ PIN (dissolved in saline containing 5% DMSO) starting the day after surgery (n = 7). Cranial bones were collected two weeks later for micro‐CT analysis and histological staining.

### LPS‐Induced Sepsis

In the sepsis model, 8‐week‐old female C57BL/6 mice were injected intraperitoneally with 2 or 10 mg kg^−1^ PIN (dissolved in saline containing 5% DMSO) for 6 h, followed by an intraperitoneal injection of LPS (20 mg/kg) (n = 7). Blood samples were collected 6 and 12 h after LPS treatment to measure serum IL‐1β, TNF‐α, and IL‐6 levels. Mice were monitored for survival for up to 72 h.

### Microcomputed Tomography (Micro‐CT) Analysis

The femurs from each group were harvested and fixed in 4% paraformaldehyde for 48 h, then scanned using a SkyScan 1176 scanner (SkyScan, Aartselaar, Belgium). The scanning parameters were set as follows: current 800 µA, voltage 50 kV, and resolution 9 µm. The region of interest (ROI) was defined as 1–4 mm proximal to the metaphyseal plate of the distal femur. The reconstructed primary parameters, BMD, BV/TV, TB. N, and TB. Sp, were analyzed using CTAn software.

### Histology and Immunohistological Staining

Mouse femurs were embedded in paraffin and sectioned into 6 µm slices. HE and TRAP staining were performed following the manufacturer's instructions. For immunohistochemistry, antigen retrieval was conducted, followed by incubation with primary antibodies Anti‐IL‐1β (1:200, ab283818) and Anti‐TNF‐α (1:200, ab307164) at 4 °C overnight. The next day, samples were incubated with the appropriate secondary antibodies for 1 h at room temperature, stained with 200 µL of DAB for 3 min, then blocked and observed under a microscope.

### ELISA

OVX mice were sacrificed at 2, 4, and 8 weeks after ovariectomy and serum was collected (n = 7). LPS‐induced sepsis mice were sacrificed 6 and 12 h after LPS treatment and serum was collected (n = 7). Cell culture supernatants were collected at 48 h after LPS and RANKL stimulation (n = 3). Mouse IL‐1β, IL‐6 and TNF‐α of serum and cell culture supernatants were determined according to manufacturer's instructions (R&D Systems).

### Cell Preparation and Stimulation

BMDMs were isolated from the bone marrow of 6‐week‐old mice and cultured for 24 h in Dulbecco's Modified Eagle's Medium (DMEM) containing 10% fetal bovine serum (FBS). The supernatants were collected, and the culture continued in DMEM supplemented with 30 ng mL^−1^ M‐CSF and 10% FBS. To induce osteoclastogenesis under inflammatory conditions, BMDMs (5 × 10⁵ cells mL^−1^) were seeded into 12‐well plates. Once reaching confluence, BMDMs were pretreated with 10, 30, and 50 ng mL^−1^ PIN for 1 h, followed by 100 ng mL^−1^ LPS and 50 ng mL^−1^ RANKL for 6 days. Afterward, TRAP staining, bone resorption assays, and total protein extraction were performed to assess the expression of NFATc1, MMP9, and CTSK.

### Cell Supernatant Protein Extraction

Cell supernatants, formaldehyde, and trichloromethane were mixed in a 4:4:1 ratio, centrifuged, and the supernatant was discarded. An equal volume of formaldehyde was then added, mixed, and centrifuged again before air drying. Finally, 80 µL of 1× SDS sample buffer was added, and the sample was mixed and boiled for 5 min prior to Western blotting.

### Assessment of Osteoclastogenesis and Bone Resorption

Assessment of osteoclastogenesis and bone resorption refer to previous studies.^[^ ^8^, ^29^ ^]^ In brief, BMDMs (5 × 10⁵ cells/well) were cultured in a 24‐well plate for 6 days using conditioned medium containing 10% FBS, 30 ng mL^−1^ M‐CSF, 50 ng mL^−1^ RANKL, and 100 ng mL^−1^ LPS. After successful osteoclast induction, TRAP staining was performed using a TRAP staining kit (Amizona Scientific, USA). The osteoclasts (OCs) were then removed from bovine bone slices using ultrasonication. Following ethanol gradient dehydration and drying by the critical point drying method, the bone slices were coated with gold in a vacuum chamber. Absorption pits were observed using an FEI Quanta 250 scanning electron microscope (Hillsboro, USA) and quantified with ImageJ software.

### ASC Oligomerisation Assays

Cells were lysed on ice with NP‐40 for 30 min. 3× SDS sample buffer was added, and the mixture was boiled for 10 min as the input. 2 mm DSS was then added to the cell lysate, mixed, and incubated for 30 min at room temperature before centrifugation. The supernatant was removed, and 3× SDS sample buffer was added again and boiled for 10 min. The samples were subsequently subjected to Western blotting.

### Mitosox

BMDMs were seeded on coverslips at a density of 2–3 × 10⁵ cells mL^−1^ and incubated overnight. The next day, the medium was replaced with DMEM containing different doses of PIN for 1 h, followed by the addition of LPS and RANKL with either MSU or ATP for 8 h. Subsequently, the cells were stained with Mitosox (5 µm) for reactive oxygen species detection. After washing the cells three times with PBS, they were fixed with 15% paraformaldehyde for 10 min at room temperature and analyzed using a Zeiss microscope.

### Intracellular K⁺ and Ca^2^⁺ Assay

The treated cell supernatant was discarded, and the cells were washed three times with potassium‐free or calcium‐free buffers, respectively. The cells were then lysed with pure HNO₃, and the lysates were collected. Nitric acid was added three times during boiling and evaporation to dryness. The dried samples were dissolved by adding ddH₂O and cooled to room temperature. Intracellular K⁺ and Ca^2^⁺ concentrations were measured using inductively coupled plasma mass spectrometry (ICP‐MS).

### Molecular Docking

The SDF format of PIN was obtained from PubChem and converted to MOL2 format using OpenBabel software. The molecular structures of NLRP3, NEK7, and ASC were retrieved from the Protein Data Bank (PDB). The NLRP3/NEK7 and NLRP3/ASC complexes were initially constructed using AlphaFold3. Molecular docking was then performed with Autodock4 to determine the affinity and binding mode of PIN to the NLRP3/NEK7 and NLRP3/ASC complexes. The complexes were optimized by removing water molecules, adding hydrogen atoms, and minimizing energy. PIN was subsequently docked into the contact regions of the complexes to identify the optimal binding site, and the results were visualized using PyMOL.

### co‐IP

For the co‐IP assay, total proteins from each group were extracted using RIPA or NP‐40. Samples were centrifuged at 4 °C for 15 min at 14000 × g, and the supernatant was immediately transferred to new EP tubes, with a portion set aside for the INPUT. Protein A+G magnetic beads were incubated for 1 h on a carousel with anti‐NLRP3, anti‐NEK7, anti‐ASC, and anti‐IgG antibodies. After magnetic separation, the corresponding NLRP3, NEK7, and ASC complexes were isolated using SDS‐PAGE loading buffer and analyzed by Western blotting.

### Cellular Thermal Shift Assay

Groups of BMDMs were lysed in RIPA buffer containing protease inhibitors. After centrifugation, the protein lysates were evenly distributed into PCR tubes. Each tube was incubated with either PIN (50 µm) or DMSO for 1 h at room temperature. The samples were then heated at different temperatures (40, 44, 48, 52, 56, 60, and 64 °C) for 3 min. After centrifugation, the supernatant was collected, and NEK7 protein levels were detected by Western blot.

### nanoDSF

NEK7 protein (1 mg mL^−1^) was mixed with pinosylvin (PIN) at a molar ratio of 1:10 and incubated on ice for 1 h. Prior to loading, the mixture was centrifuged at room temperature. The thermal stability of the protein was assessed using the Prometheus NT.48 instrument. Samples were loaded into capillaries, with each capillary drawing ten replicates. Each sample was loaded in triplicate. The temperature range was set from 20 to 95 °C with a heating rate of 1 °C per minute. Fluorescence emissions were monitored at 330 nm and 350 nm.

### Lentivirus‐Mediated Gene Overexpression and Knockdown in BMDMs

BMDMs were seeded in 12‐well plates at a density of 5 × 10⁵ cells/well. The medium was changed 48 h after LV‐NEK7 or LV‐shNEK7 transfection for subsequent cell experiments. The overexpression sequence of LV‐NEK7 was referenced from NC_000067.7 on NCBI. For knockdown of NEK7, the lentivirus sequence was 5′‐CACTGTAATGCAAGCATATAT‐3′.

### Statistical Analysis

All data were expressed as mean ± standard deviation (SD). The Shapiro‐Wilk test was used to assess the normality of the data distribution. For statistical analysis, one‐way analysis of variance (ANOVA) was applied for multiple comparisons. Comparison of the means of the two groups was performed using Student’s t‐test. Statistical calculations were performed using SPSS 25.0 (IBM, USA), with p < 0.05 considered statistically significant (*p < 0.05, **p < 0.01).

## Conflict of Interest

The authors declare no conflict of interest.

## Author Contributions

W.Z., X.W., W.L., H.Z., and Y.W. contributed equally to this work and are the first co‐authors. All authors were involved in drafting the article or revising it critically for important intellectual content, and all authors approved the final version to be published. W.Z., X.W., W.L., H.Z., and Y.W. wrote the manuscript. W.Z., X.W., W.L., J.X., and Wh.L. performed experiments and interpreted the data. W.Z., Y.Q., and Z.W. conducted animal experiments. W.Z., W.L., and G.G. recruited subjects and collected samples. S.L., L.M., and L.W. supervised the project. W.Z. supervised and designed the project. D.G. supervised, designed, and funded the project.

## Supporting information



Supporting Information

## Data Availability

The data that support the findings of this study are available from the corresponding author upon reasonable request.
