# Supplementary material for: Pinosylvin Inhibits Inflammatory and Osteoclastogenesis via NLRP3 Inflammasome
Source: Adv Sci (Weinh). 2025 Jun 10;12(31):e01532. doi: 10.1002/advs.202501532 (PMC12376636; doi:10.1002/advs.202501532)
Supplement: Supplementary file 1 — Supporting Information [file ADVS-12-e01532-s001.pdf]

ADVANCED SCIENCE
Open Access

Supporting Information

for *Adv. Sci.*, DOI 10.1002/advs.202501532

Pinosylvin Inhibits Inflammatory and Osteoclastogenesis via NLRP3 Inflammasome

Wei Zhang, Xiangbing Wu, Wenming Li, Haifeng Zhang, Yijun Wang, Jing Xu, Wenhao Li, Yi Qin, Zebin Wu, Gaoran Ge, Shujun Lv, Lu Mao*, Liangliang Wang* and Dechun Geng**

Supporting Information

Pinosylvin inhibits inflammatory and osteoclastogenesis via NLRP3 inflammasome

Authors

Wei Zhang[1,†], Xiangbing Wu[2,†], Wenming Li[1,†], Haifeng Zhang[3,†], Yijun Wang[1,†]; Jing Xu[4],Wenhao Li[1], Yi Qin[1], Zebin Wu[1], Gaoran Ge[1], Shujun Lv[5,*], Lu Mao[6,*], Liangliang Wang[7,*],Dechun Geng[1,*].

Affiliations

1 Department of Orthopaedics, The First Affiliated Hospital of Soochow University, No. 188 Shizi Street, Suzhou, Jiangsu 215006, China

2 Department of Implant Dentistry, Suzhou Stomatological Hospital, Suzhou, Jiangsu 215005, China

3 Department of Orthopaedics, Shanghai General Hospital, Shanghai Jiao Tong University School of Medicine, No.85 Wujin Road, Hongkou District, Shanghai, 200080, China

4 Department of Anesthesiology, Affiliated Hospital of Jiangsu University, 438 Jie Fang Road, Zhenjiang, Jiangsu, 212001, China.

5 Department of Orthopedics, Hai'an People's Hospital, Hai'an, Jiangsu 226000, China.

6 Department of Spine Surgery, Zhongda Hospital, Southeast University, Nanjing, Jiangsu 210009, China

7 Department of Orthopedics， the Second People's Hospital of Changzhou, the Third Affiliated Hospital of Nanjing Medical University.

†These authors contributed equally to this work.

***Corresponding authors.**

E-mail addresses:

jointlv@126.com (Shujun Lv);

101012005@seu.edu.cn (Lu Mao);

liangliangwang@njmu.edu.cn (Liangliang Wang);

szgengdc@suda.edu.cn (Dechun Geng).

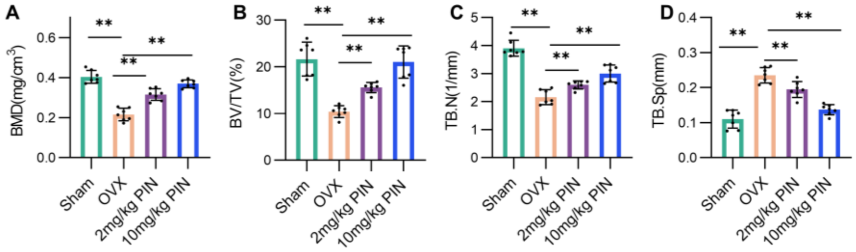

Fig.S1. PIN alleviated OVX-induced bone loss in mice. *(A-D) BMD (mg/cm^3), BV/TV (%), TB.N (1/mm), and Tb.Sp (mm) in the each group. n = 7, **p < 0.01.*

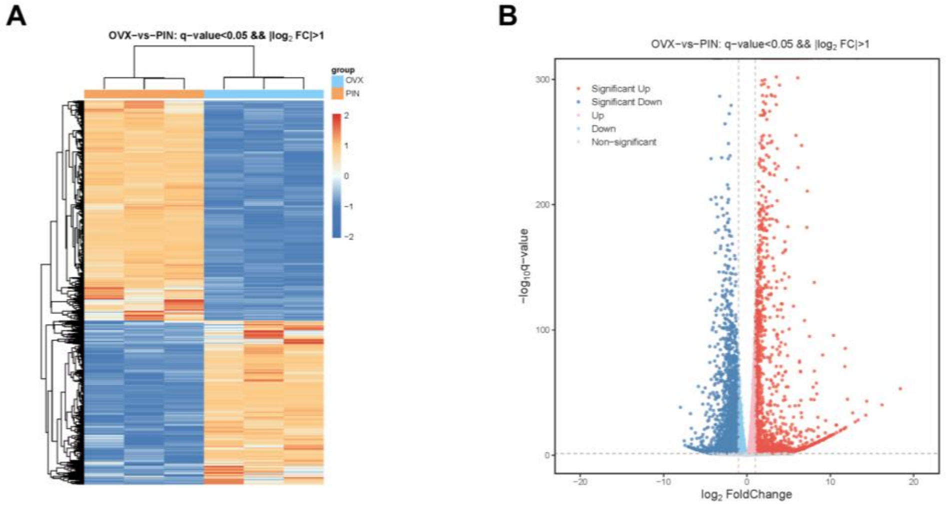

Fig.S2. RNA-sequencing of intramedullary cells in OVX and PIN mice. *(A) Heatmap of differential genes in OVX and PIN mice. (B) Volcano map of differential genes in OVX and PIN mice.*

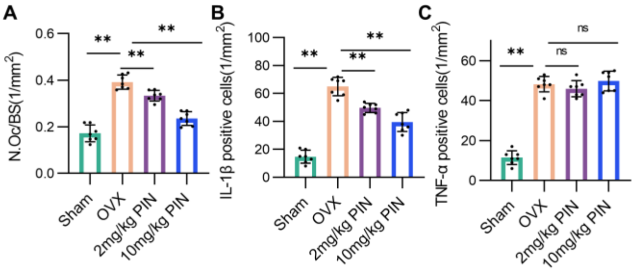

Fig.S3. PIN inhibits inflammatory and osteoclastogenesis in OVX mice. *(A) Quantitative analysis of TRAP⁺ OC in each group. n = 7. (B,C) Quantitative analysis of IL-1β⁺ and TNF-α⁺ cells in each group. n = 7. **p < 0.01, ns: p >0.05.*

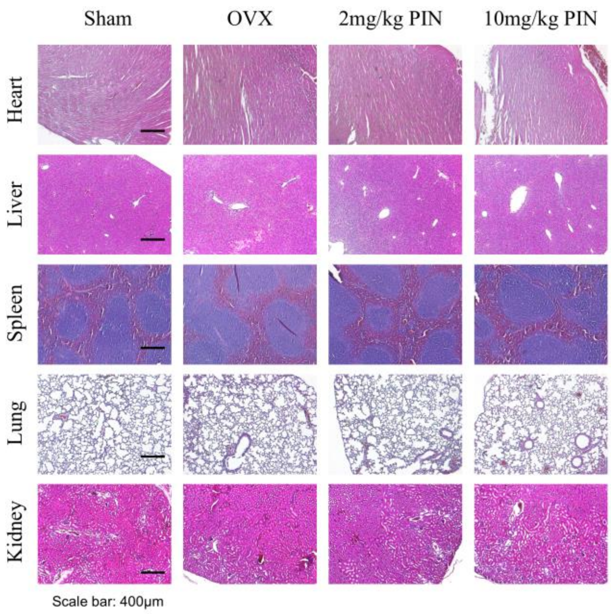

Fig.S4. Toxicity detection of PIN in mice. *HE staining of heart, liver, spleen, lung and kidney in each group. n = 7.*

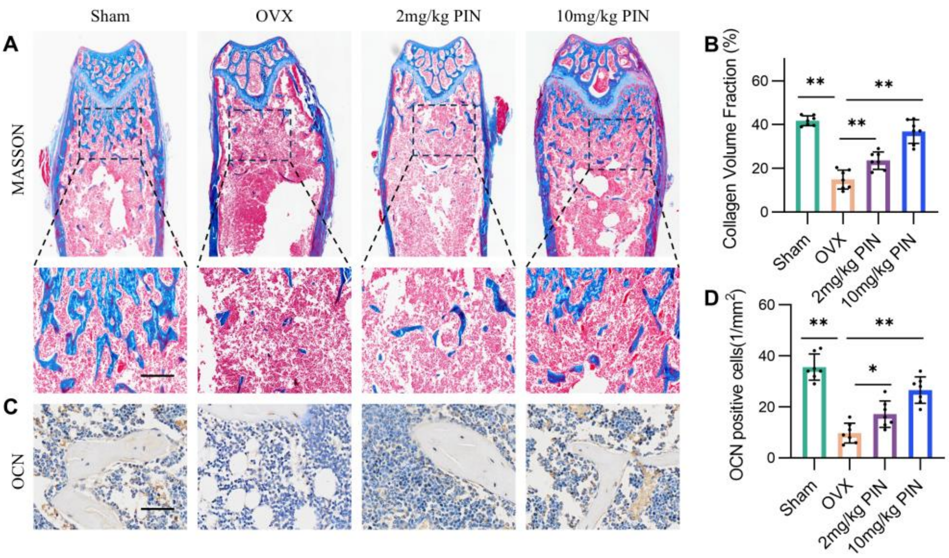

Fig.S5. PIN promoted bone formation in OVX mice. *(A, B) Representative images and quantitative analysis of MASSON staining of the femur. Scale bar = 200 μm. n = 7. (C, D) Representative images and quantitative analysis of immunohistological staining of OCN in the femur. Scale bar = 50 μm. n = 7. *p < 0.05, **p < 0.01.*

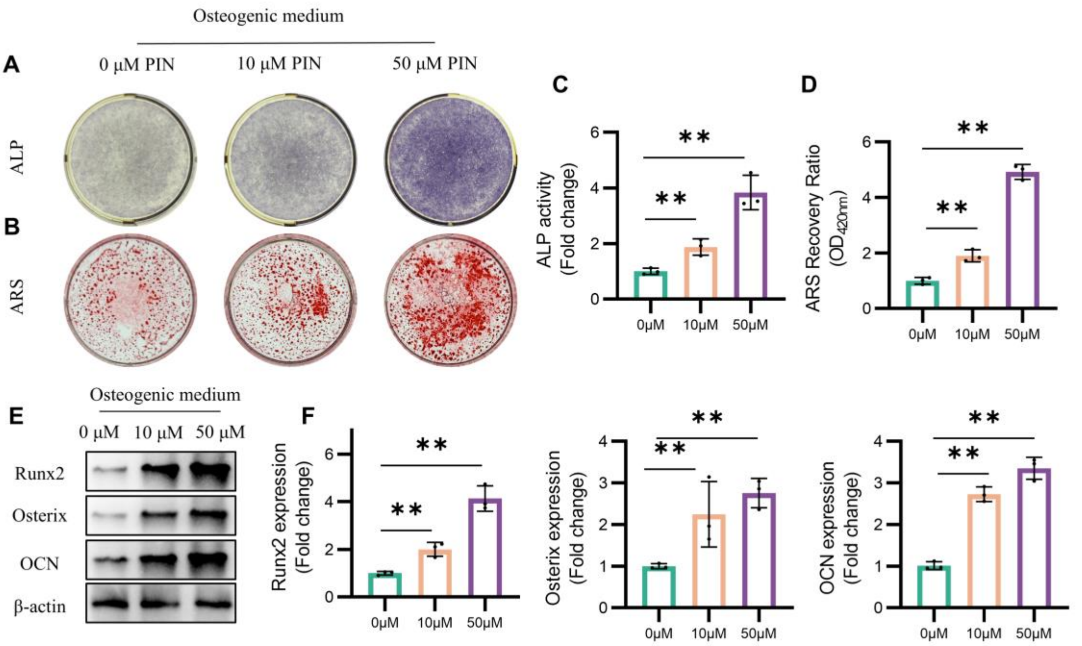

Fig.S6. PIN promoted bone formation in vitro. *(A, B) Representative images of ALP and ARS staining. (C, D) Quantitative analysis of ALP and ARS staining. n = 3. (E, F) Western blotting images and quantitative analysis of Runx2, Osterix and OCN in PIN pre-treated BMSCs which was stimulated with osteogenic medium. n = 3. **p < 0.01.*

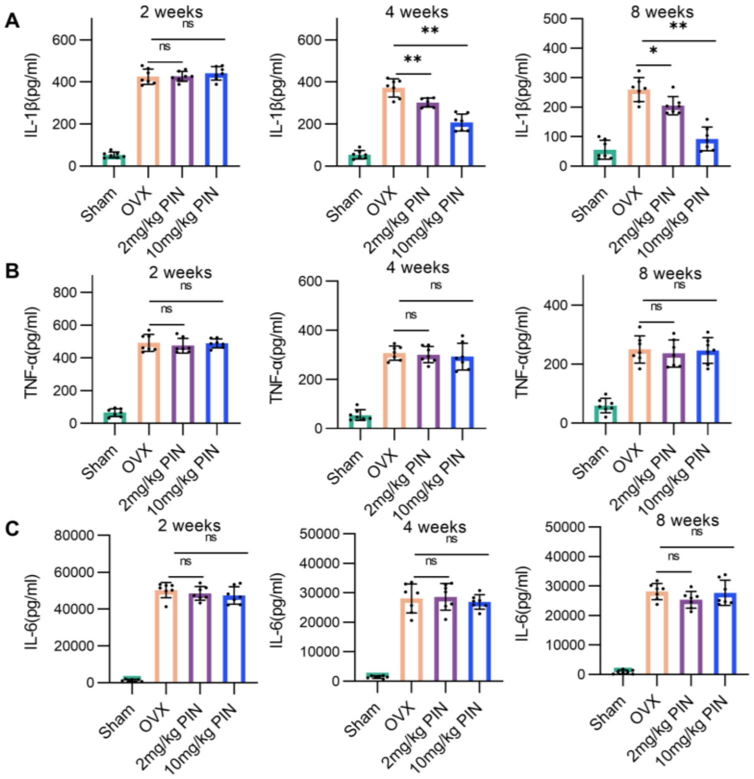

Fig.S7. PIN inhibited IL-1β serum level in OVX mice. *(A) Serum IL-1β levels at 2,4, and 8 weeks after OVX. n = 7. (B) Serum TNF-α levels at 2,4, and 8 weeks after OVX. n = 7. (C) Serum IL-6 levels at 2,4, and 8 weeks after OVX. n = 7. *p < 0.05; **p < 0.01; ns: p >0.05*

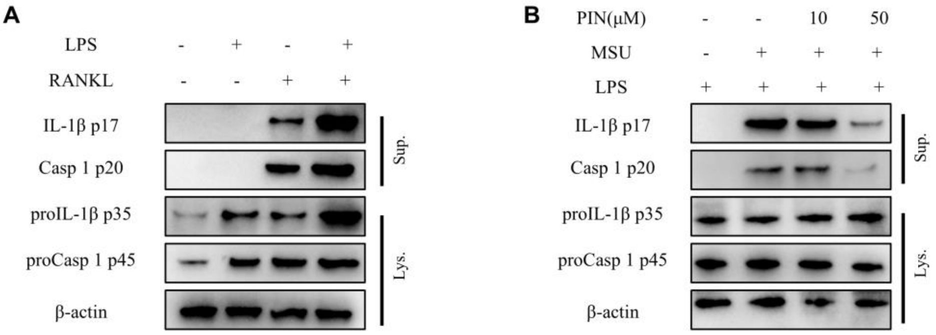

Fig.S8. LPS+RANKL induced cleavage of IL-1β and Casp 1, and PIN block cleavage of IL-1β and Casp 1 stimulated by LPS+MSU in macrophages. *(A) BMDMs were stimulated with LPS and RANKL for 24 h. Cell lysates and supernatants were analysed by immunoblotting. n = 3. (B) BMDMs were treated with PIN for 1h, and then stimulated with LPS and MSU. Cell lysates and supernatants were analysed by immunoblotting. n = 3.*

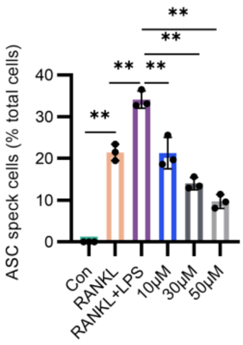

Fig.S9. PIN block ASC assembly stimulated by LPS and RANKL in macrophages. *Quantitative analysis of ASC speck cells in each group. n = 3. **p < 0.01.*

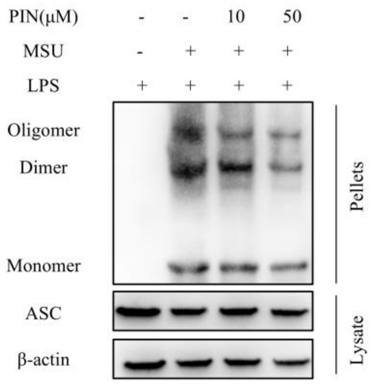

Fig.S10. PIN block ASC assembly stimulated by LPS and MSU in macrophages. *Representative immunoblotting images of ASC oligomerization in cross-linked cytosolic pellets. n = 3.*

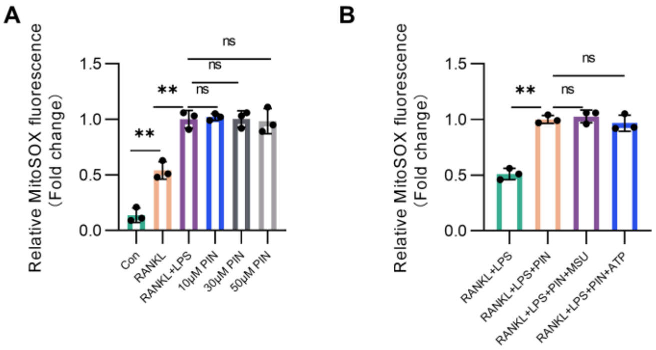

Fig.S11. The inhibitory effect of PIN on NLRP3 inflammasomes is independent of mitochondrial oxidative stress. *(A, B) Quantitative analysis of MitSOX in each group. n = 3. ns: p >0.05.*

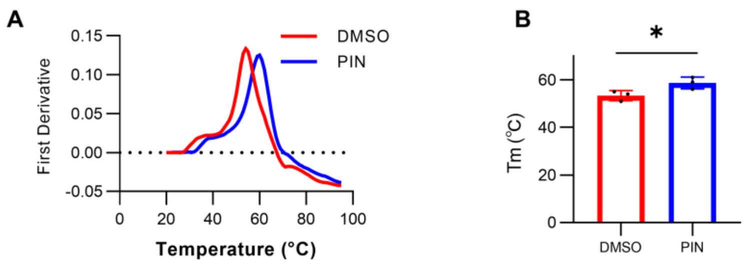

Fig.S12. The influence of PIN on the thermal stability of NEK7. *(A, B) The nanoDSF analysis of PIN and NEK7. n = 3. *p < 0.05.*

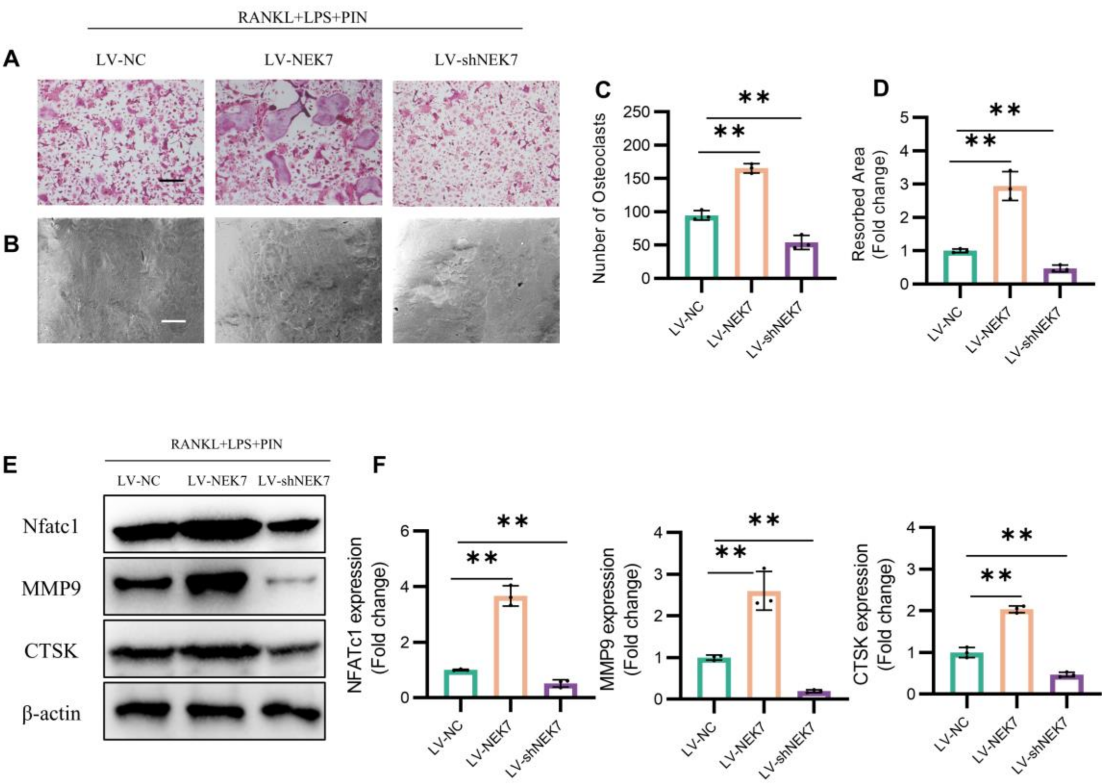

Fig.S13. LV-NEK7 reversed PIN-induced osteoclastogenesis triggered by LPS and RANKL. *(A) TRAP staining to evaluate the effect of LV-NEK7 and LV-shNEK7 on the size of OCs. Scale bar = 200 μm. n = 3. (B) Bone plate resorption assay to evaluate the effect of LV-NEK7 and LV-shNEK7 on the bone resorption of OCs. Scale bar = 50 μm. n = 3. (C) Quantitative analysis of the number of OCs in TRAP staining. n = 3. (D) Quantitative analysis of bone resorption in each group. n = 3. (E, F) Western blotting images and quantitative analysis of NFATc1, MMP9 and CTSK in LV-NEK7 or LV-shNEK7-treated OCs. n = 3. **p < 0.01.*

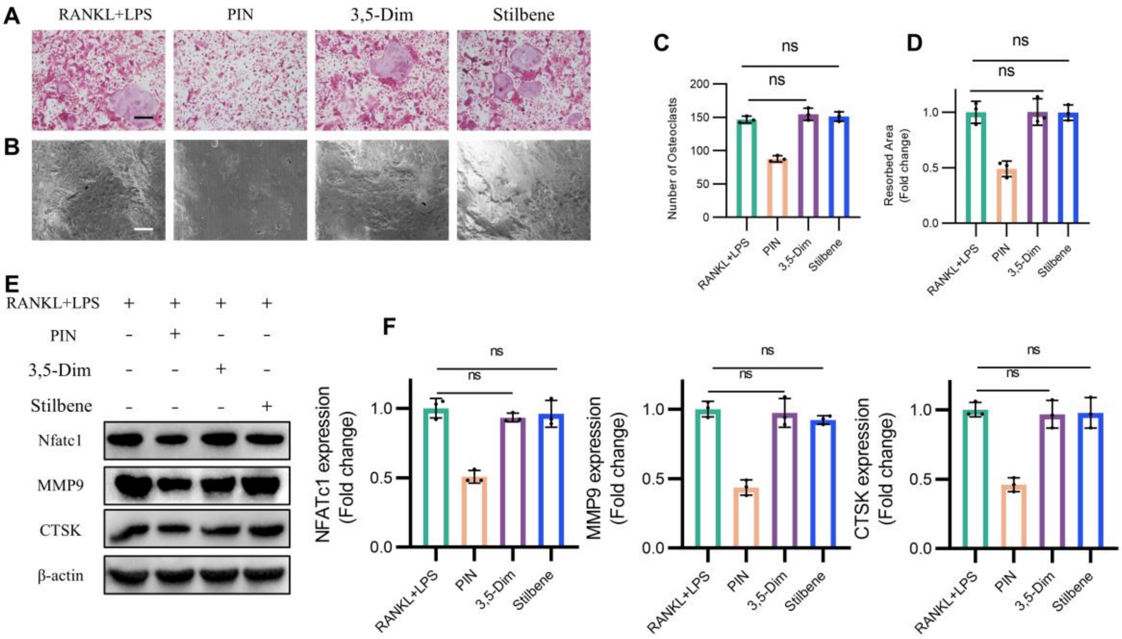

Fig.S14. Unlike pinosylvin, 3,5-dimethoxystilbene and stilbene do not inhibit osteoclastogenesis. *(A) TRAP staining to evaluate the effect of 3,5-dimethoxystilbene and stilbene on the size of OCs. Scale bar = 200 μm. n = 3. (B) Bone plate resorption assay to evaluate the effect of 3,5-dimethoxystilbene and stilbene on the bone resorption of OCs. Scale bar = 50 μm. n = 3. (C) Quantitative analysis of the number of OCs in TRAP staining. n = 3. (D) Quantitative analysis of bone resorption in each group. n = 3. (E, F) Western blotting images and quantitative analysis of NFATc1, MMP9 and CTSK in 3,5-dimethoxystilbene and stilbene -treated OCs. n = 3. ns: p >0.05.*

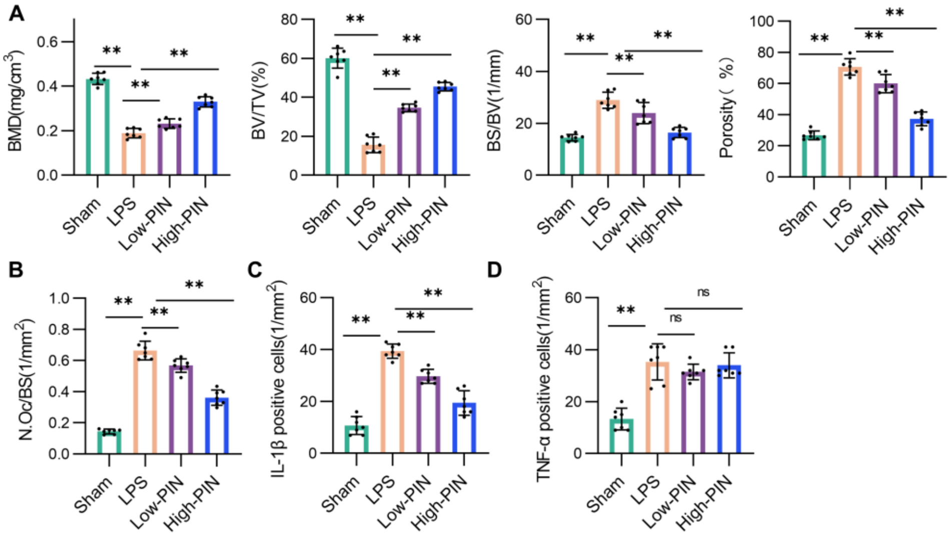

Fig. S15. PIN alleviates the process of osteolysis. *(A) BMD (mg/cm^3), BV/TV (%), BS/BV (1/mm), and porsity in each group. n = 7. (B) Quantitative analysis of TRAP$^+$ OC in each group. n = 7. (C, D) Quantitative analysis of IL-1β$^+$ and TNF-α$^+$ cells in each group. n = 7. **p < 0.01; ns: p >0.05.*
